# Public Acceptability of E-Mental Health Treatment Services for Psychological Problems: A Scoping Review

**DOI:** 10.2196/mental.6186

**Published:** 2017-04-03

**Authors:** Jennifer Apolinário-Hagen, Jessica Kemper, Carolina Stürmer

**Affiliations:** ^1^ Institute for Psychology Department of Health Psychology University of Hagen Hagen Germany; ^2^ Institute for Psychology University of Vienna Vienna Austria

**Keywords:** mental health, eHealth, acceptability of healthcare, public opinion, attitude to computers, patient preference, diffusion of innovation, cognitive therapy, computer literacy, review

## Abstract

**Background:**

Over the past decades, the deficient provision of evidence-based interventions for the prevention and treatment of mental health problems has become a global challenge across health care systems. In view of the ongoing diffusion of new media and mobile technologies into everyday life, Web-delivered electronic mental health (e-mental health) treatment services have been suggested to expand the access to professional help. However, the large-scale dissemination and adoption of innovative e-mental health services is progressing slowly. This discrepancy between potential and actual impact in public health makes it essential to explore public acceptability of e-mental health treatment services across health care systems.

**Objective:**

This scoping review aimed to identify and evaluate recent empirical evidence for public acceptability, service preferences, and attitudes toward e-mental health treatments. On the basis of both frameworks for technology adoption and previous research, we defined (1) perceived helpfulness and (2) intentions to use e-mental health treatment services as indicators for public acceptability in the respective general population of reviewed studies. This mapping should reduce heterogeneity and help derive implications for systematic reviews and public health strategies.

**Methods:**

We systematically searched electronic databases (MEDLINE/PubMed, PsycINFO, Psyndex, PsycARTICLES, and Cochrane Library, using reference management software for parallel searches) to identify surveys published in English in peer-reviewed journals between January 2010 and December 2015, focusing on public perceptions about e-mental health treatments outside the context of clinical, psychosocial, or diagnostic interventions. Both indicators were obtained from previous review. Exclusion criteria further involved studies targeting specific groups or programs.

**Results:**

The simultaneous database search identified 76 nonduplicate records. Four articles from Europe and Australia were included in this scoping review. Sample sizes ranged from 217 to 2411 participants of ages 14-95 years. All included studies used cross-sectional designs and self-developed measures for outcomes related to both defined indicators of public acceptability. Three surveys used observational study designs, whereas one study was conducted as an experiment investigating the impact of brief educational information on attitudes. Taken together, the findings of included surveys suggested that e-mental health treatment services were perceived as less helpful than traditional face-to-face interventions. Additionally, intentions to future use e-mental health treatments were overall smaller in comparison to face-to-face services. Professional support was essential for help-seeking intentions in case of psychological distress. Therapist-assisted e-mental health services were preferred over unguided programs. Unexpectedly, assumed associations between familiarity with Web-based self-help for health purposes or “e-awareness” and intentions to use e-mental health services were weak or inconsistent.

**Conclusions:**

Considering the marginal amount and heterogeneity of pilot studies focusing on public acceptability of e-mental health treatments, further research using theory-led approaches and validated measures is required to understand psychological facilitator and barriers for the implementation of innovative services into health care.

## Introduction

Many individuals with mental health problems do not receive prompt professional support delivered face-to-face in health care in times of need. More and more persons thus tend to seek help for mental health purposes on the Internet. Limited resources of health care units as global key issue for the large-scale dissemination of interventions for the prevention and treatment of common mental health problems thus require innovative strategies. Given the persisting problem of treatment gaps in mental health care, providing effective treatments via the Internet has been suggested as a cost-efficient way to expand public access to mental health services on a large scale [1-3].

Web-based and computerized, respectively electronic mental health (e-mental health) services use new media and innovative digital technologies to provide screening, psychoeducation, health promotion, prevention, self-help, counseling, therapy, and aftercare [3-6]. Evidence-based e-mental health treatments are available for mental health problems concerning mood [7,8], anxiety [9-11], substance abuse [12], and eating disorders [13]. Delivery modes and treatment formats vary from unguided Web-based self-help treatments services to therapist-guided e-mental health treatments. In controlled trials, especially therapist-guided, Internet-based cognitive behavior therapy (iCBT) approaches achieved effect sizes, satisfaction, and adherence rates comparable to those of traditional face-to-face CBT [14]. However, poor engagement of primary care patients [15-17] and the slow diffusion of e-mental health into mental health care indicated acceptability issues as barrier for the dissemination of e-mental health treatments [2,6]. This outlined discrepancy between promising research findings and the weak uptake of e-mental health treatments in real-world help-seeking contexts needs clarification about facilitators and barriers of successful dissemination of e-mental health [6]. While facilitators of Web-based treatments’ uptake such as the specific role of professional support in therapeutic outcomes [18,19] and the familiarity with Web-based media [20] are discussed, predictors of seeking help outside the context of clinical trials largely remain unclear. This outlined discrepancy between research and practice makes it thus necessary to identify indicators of public acceptability of e-mental health [6].

Previous research on acceptability of digital health interventions has emphasized the central role of profoundly understanding the views and needs of persons using digital health interventions [21]. For this purpose, theoretical frameworks appear applicable to investigate public acceptability of e-mental health treatments. For instance, the diffusion of innovation theory [22] aims to explain how the dissemination and adoption of innovative technologies develops from a sociological perspective. However, this theory deals with complex developments over a period. From a psychological viewpoint, as stated in this paper, technology acceptance models (TAM) appear better applicable. TAM provide an empirically grounded framework to understand facilitators and barriers of individual intentions to use and the adaption of information technology (IT) [23]. The “Unified Theory of Acceptance and Use of Technology” (UTAUT) [24] is an expansion of the original TAM-framework, which is based on elements of eight models developed in IT acceptance, psychological, and sociological research, such as the diffusion of innovation theory [22]. To examine determinants of technology use and behavioral intentions to use, the UTAUT provides different key determinants of IT acceptance and moderators such as age, gender, and experience. UTAUT research showed that the determinant “performance expectancy” that includes the domains “perceived usefulness,” “relative advantage,” “outcome expectations,” and “extrinsic motivation” is the best predictor for IT acceptance in terms of intentions to use [24,25].

Over the past decades, the utility of the UTAUT has been confirmed in several IT-driven organizational contexts, such as the adoption of eHealth by health care providers [26]. Although the UTAUT is still rarely cited in research targeting the uptake of e-mental health treatments, some components of the framework can be found in recent surveys, such as perceived usefulness (helpfulness) and intentions to use e-mental health. In addition, most studies investigated preferences and attitudes toward e-mental health services not in the general population, but within specific populations, including adolescents [27-30], patients [31,32], and health professionals [33,34]. Furthermore, various nonclinical surveys targeted Web-based self-help and health information. For instance, Oh et al [27] explored user preferences and perceptions of helpfulness of self-help websites among young Australians. Given the impact of moderators as stated in the UTAUT [24], these findings obtained from selected target groups appear barely transferable to public acceptability of e-mental health treatments.

Hence, to predict psychological facilitators and barriers of the large-scale uptake of e-mental health treatments in public health there is need to look at the findings of surveys examining public options about these innovations with samples consisting of a diverse cross-section of the general population (in terms of data collection in the respective regional context of individual studies). Yet, public opinions about e-mental health treatments have been scarcely considered in earlier stages of e-mental health development. Previous reviews have thus mainly targeted the evidence base for the effectiveness of Web-based therapies for diagnosed mental disorders [7-14] and Web-based self-help formats [35]. Other types of existing e-mental health reviews focused on proposed relative advantages and challenges for mental health care [1-3,17]. Considering the above-outlined divide between the overall good satisfaction or acceptability of participants in controlled trials and the low impact in health care across the globe [2,3,6,17], a scoping review targeting the “status quo” of public acceptability of e-mental health through the identification of potential indicators of acceptability (ie, perceived helpfulness and intentions to use) can offer first insights into the “black box” of prospective service users. By this means, a scoping review can help derive implications for both outcomes in systematic reviews and strategies in public health initiatives that aim to better meet (information) needs of a broad range of citizens.

Therefore, the purpose of this scoping review was to determine the international “status quo” of public acceptability of e-mental health treatment services across different health care systems. On the basis of previous work [23,24], both (1) perceived helpfulness and (2) intentions to use in case of future mental health problems (likelihood of future use) were chosen as potential indicators for public acceptability of e-mental health treatment services. In the UTAUT, perceived usefulness (helpfulness) is a component of performance expectancy affecting intentions to use, which in turn predict actual usage (adoption). The term “public” refers to the general population of the country or region where research has been conducted. To make meaningful interpretations, surveys eligible for inclusion in this scoping review need to contrast both indicators for at least two distinctive provision modes of mental health treatment services (relative advantage; eg, preference of Web-based vs traditional services, guided vs unguided Web-based programs). Another purpose of this mapping was to reduce heterogeneity of results, which should allow implications for research questions in future reviews and public health strategies. Accordingly, we addressed the following research questions:

1. Perceived helpfulness of e-mental health treatments: Do persons recruited from the general population perceive e-mental health treatment services as helpful treatment options in case of (impeding) emotional problems? To answer this question, two subquestions with comparators (preference) were formulated: Are there mental health service type-specific differences in the assessment of the perceived helpfulness, depending on (1a) the delivery mode (Web-based vs face-to-face) or (1b) the provision of professional guidance (unguided vs guided e-mental health services)?

2. Intentions to use e-mental health treatments: To what extent are persons recruited from the general population willing to use e-mental health treatments in case of emotional problems? In other words, are there mental health service type-specific differences in intentions to use e-mental health treatment services, depending on (2a) the delivery mode (Web-based vs face-to-face) or (2b) the provision of professional guidance (unguided vs guided e-mental health services)?

## Methods

### Overview

The conduction and reporting of this scoping review refers to the preferred reporting items for systematic reviews and meta-analyses (PRISMA) guidelines [36], as far as applicable. Due to the focus of this scoping review, we could not apply all of the 27 items suggested by the PRISMA consortium. As mentioned by Liberati et al [36], the PRISMA statement is designed for systematic reviews and meta-analyses of randomized controlled trials (RCTs) and is not fully applicable to other types of reviews of health research. Thus, we do not report these items of the PRISMA checklist: data collection process, summary of measures, synthesis of results, and risk of bias across studies (eg, gray literature) and additional analyses.

### Eligibility Criteria

Eligible studies (1) recruited participants from the general population, (2) were conducted outside the context of clinical studies, (3) compared public views about different provision modes of mental health treatment services, (4) scoped on indicators of public acceptability of e-mental health treatments (perceived helpfulness and intentions to use), and (5) used cross-sectional (quasi-) experimental study designs.

#### (P) Populations

This review included articles targeting a broad range of participants from the general population (representative or convenience samples with participants over the age of 14 years). As we reviewed international research, the definition of general population depended on the region of data collection in individual studies.

#### (I) Interventions

This scoping review was concerned with observational surveys on the assessment of public views on e-mental health conducted outside the context of diagnostic, psychosocial, or therapeutic interventions. Experiments could be considered. Interventions (e-mental health treatments) assessed in surveys were fictional.

#### (C) Comparators

Studies were included in this review if they aimed to assess public acceptance, expectations, or attitudes toward e-mental health services in case of emotional distress (comparisons of at least two provision modes were obligatory, differing in delivery modes and/or professional support = relative advantage). For instance, eligible studies compared public opinions about (1) e-mental health and face-to-face treatments and/or (2) guided and unguided e-mental health treatments.

#### (O) Outcomes

As outlined before, we defined indirect individual determinants (indicators) for public acceptability of e-mental health treatments: (1) perceived helpfulness and (2) intentions to future use e-mental health services in case of emotional distress. In addition, we aimed to explore factors explaining variance in public attitudes, such as IT user experience.

#### (S) Study Designs

Eligible surveys included quantitative data collection through questionnaires in cross-sectional observational or experimental studies.

### Information Sources

We systematically searched electronic databases (MEDLINE/Pubmed, PsychARTICLES, PSYNDEX, PsycInfo, and Cochrane Library) to identify empirical surveys scoping on indicators of public acceptability of e-mental health treatments published in peer-reviewed journals between January 2010 and December 2015. We chose these specific medical and psychological databases because our review focused on psychological aspects of e-mental health uptake. We limited the time span to the five years of diffusion (2010-2015) because the diffusion of innovation is complex, varying across settings and periods [22]. This decision was also based on previous experience with database searches for literature regarding this research field (Multimedia Appendix 1). As a result of this earlier work, we did not search for studies published before 2010 to reduce heterogeneity of results for this scoping review (given the assumed stage of familiarity with new media, diffusion of mobile phone–delivered mobile Internet, and advances in e-mental health). The last date we searched databases and additional sources for literature was the February 15, 2016.

### Search

We used the following keywords for database searches: “e-mental health” AND (“attitude” OR “acceptability” OR “acceptance” OR “preference” OR “perception” (Boolean operator). We used the commercial reference management software Citavi (Swiss Academic Software, Switzerland) to conduct the searches in electronic databases simultaneously (to avoid duplicates and irrelevant records). In other words, we searched all databases together in one step (using the same keywords for each database). Due to the novelty of the distinct research field of interest, we included the keyword “e-mental health” that is not listed in medical subject headings (MeSH terms). For additional searches via ResearchGate and Google Scholar, we used further keywords, such as “online self-help,” “online therapy,” “Internet-based psychotherapy,” and “iCBT.” Furthermore, we screened bibliography lists of papers identified through electronic databases. In this scoping review, we did not search the gray literature for publication bias.

### Study Selection

The study selection process involved different stages and reviewers (CS, JA, and JK).

#### Step 1: Systematic Review (Previous Work)

Two reviewers (CS, JA) searched databases independently in December 2015 (CS), January (CS, JA), and February 2016 (JA). JA and CS independently searched and reviewed the literature from January 2005 to December 2015 for articles scoping on public attitudes toward e-mental health published in both English and German peer-reviewed journals (separate database searches). The search strategy was broad and included all age groups, study designs, and subgroups outside of clinical trials (professionals, clients, risk groups). The search was monitored and cross-checked by a third investigator (JK). CS reported the results of 24 reviewed studies in her Bachelor’s thesis (completed in April 2016). Key findings of this review were presented as poster at the Fourth European Society for Research on Internet (ESRII) conference in September 2016 in Bergen, Norway (Multimedia Appendix 1).

#### Step 2: Scoping Review

On the basis of key findings of this initial review (Multimedia Appendix 1), a second search for this scoping review was conducted using Citavi (JA, JK) and compared with previous results obtained in the first stage (JA, CS). In case of inconsistencies, JK decided on study selection to achieve consensus (April 2016).

The search strategy we used in February 2016 involved a parallel database search to avoid duplicates and irrelevant records for the scoping review. Database searches were conducted mainly in January 2016. Last searches for additional studies were conducted in February 2016. For the selection process, two reviewers (CS, JA) independently conducted a broad study search and selection procedure for the systematic review (January 2005 to December 2015) and a narrowed search strategy to reduce heterogeneity of records via parallel searches (January 2010 to December 2015) for the scoping review (JA, JK). After reading the titles and abstracts of records, we screened full-texts for meeting the inclusion criteria of this scoping review. Studies were scoped on predefined indicators of public acceptability of e-mental health treatment services. Consequently, we excluded surveys with data collected in efficacy or feasibility studies, clinical RCTs, reviews, or case studies. Studies targeting specific groups (eg, adolescents), qualitative surveys, and articles published in other than English language were excluded, too. Key findings of this scoping review were presented as poster at thirtieth European Health Psychology Society (EHPS) conference in August 2016 in Aberdeen, Scotland (Multimedia Appendix 2).

### Data Items

As noted above, the rationale of this scoping review was based on findings of a previous systematic review (see Multimedia Appendix 1). Research questions were developed using the PICOS approach as suggested by the PRISMA statement [36]: (P) populations, (I) interventions, (C) comparators, (O) outcomes, and (S) study designs (for details, see the Eligibility Criteria subsection). Indicators of public acceptability were perceived helpfulness and intentions to use (likelihood of future use) e-mental health treatment services.

### Risk of Bias in Individual Studies (Study Quality Assessment)

JA and JK evaluated the study quality with an adapted version of the Newcastle-Ottawa scale (NOS) [37] for cross-sectional studies by Herzog et al [38]. The NOS consists of three categories with different rating dimensions for quality assessment. The first category, “selection” (maximum 5 of 10 stars), includes (1) “representativeness of the sample” (four rating options, maximum 1 star), (2) “sample size” (two options, maximum 1 star), (3) “non-respondents” (three options, maximum 1 star), and (4) “ascertainment of the exposure (risk factor)” (three options, maximum 2 stars). The second category, “comparability” (maximum 2 of 5 stars), includes “assessment of control variables” (two options, maximum 2 stars). The third category, “outcome” (maximum 3 of 10 stars), consists of (1) “assessment of the outcome” (four options, maximum 2 stars) and (2) “statistical test” (two options, maximum 1 star). As information on the scoring (cut-off thresholds; poor-fair-good) of the NOS is under development for observational nonrandomized studies [37], we did not refer to NOS scoring algorithms suggested for quality assessments of RCTs because they were hardly convertible. Instead, we developed the following heuristics for thresholds (maximum 10 stars): 0-3 stars (poor quality), 4-6 stars (fair quality), and 7-10 stars (good quality). Due to the novelty of the scoped research field (mainly pilot studies), we assessed studies as fair in their quality if the categories “selection” and “outcome” were both assigned with at least two stars per study. Because we focused on (nonrandomized) surveys, we found it problematic to define stars for “comparability” obligatory for the quality assessment.

## Results

### Study Selection

**Figure 1:**
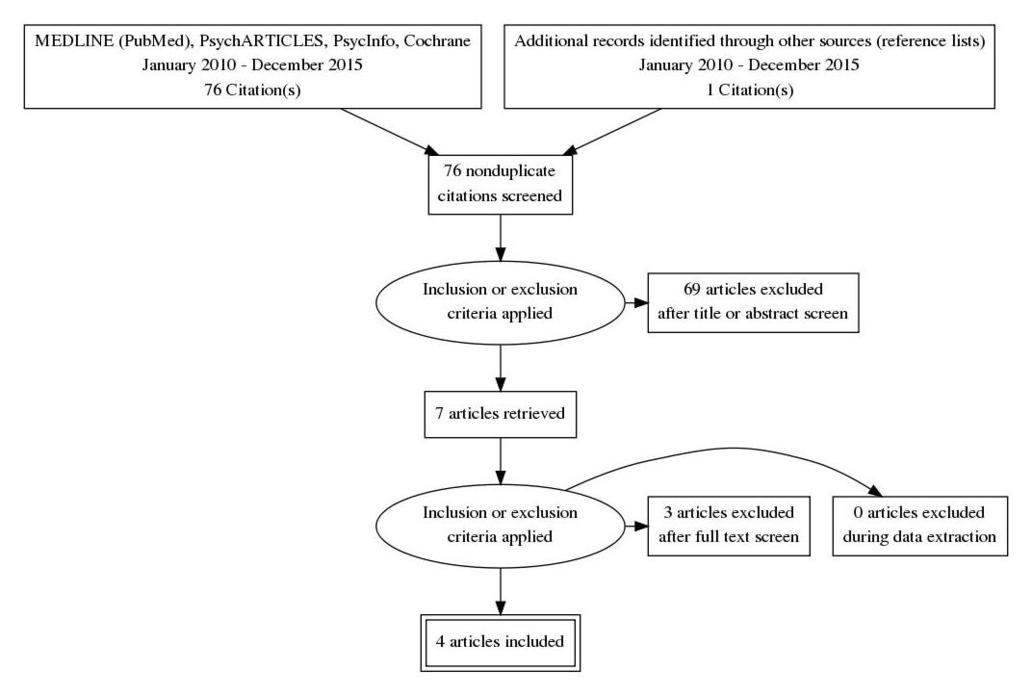
PRISMA (preferred reporting items for systematic reviews and meta-analyses) flow diagram of study selection.

In total, we identified 76 records through parallel database searches. Of initially 77 records, one duplicate was removed. Figure 1 illustrates the study selection procedure. After removing 13 records through their title, we screened 63 abstracts. We excluded 56 records. Removed publications were reviews, effectiveness and feasibility RCTs, studies with clinical scope, case studies, or surveys out of our scope in terms of population, measured constructs, or interventions. After obtaining and reading seven full-texts, we finally excluded further three papers [39-41], which did not have their scope on the predefined indicators of public acceptability. Finally, we included four publications [6,42-44] in this scoping review. One included paper [42] was obtained from the reference list of another article [43]. We achieved consensus regarding the study selection for this scoping review (JA, JK, CS).

### Study Characteristics

Table 1 summarizes the study characteristics and central findings of the four included studies published between 2010 and 2014.

**Table 1:** Summary of study characteristics and main findings.

Study	Design	Aims	Sample^a^	Method and instruments^b^	Main findings
Klein and Cook [42]^c^	Cross-sectional online survey	To identify differences between “e-preferers” and “non e-preferers” on the perceived helpfulness and intentions to use e-mental health in comparison to traditional services	Online sample (N=218) of the Australian general population. Age range = 18-80 years; Mean 36.6 (SD 14.5) years Female (75.7%) “*e-prefers” (n=50); “non e-prefers” (n=168) 63.9% with mental health service experience*	Self-developed online survey “e-preference” (grouping condition) Perceived helpfulness of 11 mental health services (helpful, neither, harmful) Concerns using e-mental health Likelihood of future using mental health services (intentions to use) Four validated measures on self-stigmatization (DDS), locus of control or LOC (MHLC-C), big-five personality traits (TIPI), and learning style (VARK) Response rate (completed survey): 91.3% (n=199)	Preference toward using traditional to e-mental health services (77.1% were “non e-preferers”) “e-preferers” were more willing to use and assess e-mental health services as more helpful Previous experience with mental health services included psychologists (84.2%), information websites (71.2%) “non e-preferers” were more concerned about confidentiality “e-preferers” scored higher on self-stigma and chance LOC; “non e-preferers” scored higher on emotional stability and doctor LOC (for instance)
Casey et al [43]^d^	Cross-sectional online RCT^e^ (mixed factorial design)	To determine the impact of educational information on attitudes (ie, perceived helpfulness and intentions to use different e-mental health services	Online sample (N=217) of the Australian general population. Age range = 17-60 years; Mean 29.7 (SD 11.9) years Female (78%) *groups: text (n=66), film (n=72), control (n=70)*	Self-developed online survey, a modified version of another measure [42] Perceived helpfulness of four e-mental health services (online counseling, information websites, and online program with or without therapist contact) Likelihood of future using e-mental health services (intentions to use) Concerns regarding the usage of e-mental health Random assignment of respondents to one of three conditions (text intervention, film intervention (2.5 minutes), or no intervention or control condition)	Preference toward using e-mental health services with therapist assistance The likelihood of using e-mental health services was improved in the text condition group, but not in the film condition group Neither the text- nor video-based information affected the perceived helpfulness of e-mental health in comparison to the control condition
Eichenberg et al [44]^f^	Cross-sectional survey (panel interviews)	To explore public media use, the perceived impact of health information sources, and the intentions to use e-mental health in comparison to traditional services (for anxiety)	Representative sample (N=2411) of the German general population Age range = 14-90 years; Mean 51.0 (SD 18.6) years Female (53.2%) *41% never used computers*	Self-developed survey (pretest for with n=67) Public media use, preferred sources of health information, and their impact on health behavior Use of and willingness to use psychological online counseling, and media-assisted therapy in comparison to traditional face-to-face mental health services in case of emotional distress	Preference toward using traditional to e-mental health services Previous use of the Internet for health information was associated with a higher willingness to use online counseling Sociodemographic data (eg, younger age, female gender, higher education) and Internet usage corresponded with intentions to use e-mental health
Musiat et al [6]^g^	Cross-sectional Web-based survey	To explore acceptability of e- and m-mental health services in comparison to traditional services (attitudes and expectation, and intentions to use)	Web-based sample (N=490) of the English general population Age range =18-78 years; Mean 26.7 (SD 8.9) years Female (78.2%) *49% with history of mental health problems, 22% with current mental health issues*	Self-developed survey (12 important domains were grounded on ratings of a focus group of service users) Previous and current psychological problems, help-seeking behavior, and computer literacy Expectations, attitudes, and acceptability ratings: importance of domains of mental health services Perceived benefits and likelihood of future use of e-mental health and m-mental health in comparison to traditional face-to-face therapy and self-help books	Preference toward using traditional to e-mental health programs and m-mental health apps Face-to-face treatments were most likely to meet respondents’ expectations in most important domains (eg, helpfulness, credibility) Overweight of negative attitudes and expectations about e-mental health and m-mental health self-help services mHealth apps had the lowest acceptability ratings when compared with other mental health services

^a^Noteworthy features of the sample are shown in italics. All surveys included in this scoping review reported sociodemographic information and had informed consent as inclusion criteria for participation.

^b^Measures used by [42]: DDS=devaluation discrimination scale; MHLC-C=multidimensional health locus of control scales, form C; VARK=VARK learning styles inventory; TIPI=ten-item big-five personality inventory.

^c^Convenience sample from Australia.

^d^Convenience sample from Australia.

^e^RCT: randomized controlled trial.

^f^Panel interviews from Germany.

^g^Convenience sample from England.

#### Participants and Study Characteristics

Samples of the general population were surveyed in two studies from Australia [42,43], one study from Germany [44], and another study from England [6]. One study collected data via panel interviews in cooperation with a market research institute [44]. The other three studies were Web-based surveys recruiting participants through social media websites, e-mail, flyers, and undergraduate courses [6,42,43]. Sample sizes ranged from 217 to 2411 respondents (age span: 14-95 years). The oldest included publication by Klein and Cook [42] examined whether individual differences exist between persons who prefer e-mental health services and those who prefer traditional services among an Australian Web-based sample (n=218). The second included study by Casey et al [43] investigated whether brief text- or film-based educational interventions improve attitudes toward e-mental health services among an Australian Web-based sample (n=217). The third included study by Eichenberg et al [44] explored the use of public media for mental health purposes among a representative sample of the German general population (n=2411). The fourth included study by Musiat et al [6] explored public acceptability of e-mental health and mobile (mHealth) self-help treatment services among a British Web-based sample (n=490).

#### Interventions

Fictional e-mental health treatment services described for acceptability assessments. Klein and Cook [42] investigated perceived helpfulness and the intentions to use 11 different traditional and e-mental health services, which included general practitioner (GP), psychologist, psychiatrist, counselor, self-help book, information website, Web-based or telephone counseling, Internet-based program with or without therapist assistance, and prescribed medication. The RCT by Casey et al [43] was conducted using a modified version of another measure [42] with four e-mental health services (therapist-assisted e-mental health treatments, unguided e-mental health treatments, information websites, and online counseling). Study participants were randomly assigned to either one of two experimental conditions (film- or text-based information) or the control group. The educational material contained information about e-mental health. In the film condition, a 25-year-old male read the text (two and a half minutes) [43]. Eichenberg et al [44] explored the impact of health information sources (eg, GP, psychologist, and websites), awareness and use of online counseling, and intentions to use mental health services (face-to-face and Web-based or virtual reality treatments). Musiat et al [6] asked their respondents to indicate whether the four mental health services meet their expectations (face-to-face therapy, self-help books, and both e-mental health and mHealth self-help treatment services).

#### Comparators

To investigate individual differences between “e-preferers” and “non e-preferers,” Klein and Cook [42] used four validated measures on stigma perception, locus of control, learning styles, and “Big-5” traits. Casey et al [43] compared attitudes between those who received psychoeducation and the control group. Group comparisons in the study by Eichenberg et al [44] involved experience with using the Internet for mental health purposes and sociodemographic differences. The survey by Musiat et al [6] compared differences in computer literacy, demographic background, and history of mental health problems.

#### Outcomes

Klein and Cook [42] used both self-developed survey and four validated measures. The self-developed survey included perceived helpfulness and intentions to use 11 mental health services. Participants in the RCT by Casey et al [43] were asked to indicate perceived helpfulness and intentions to use four e-mental health services. Participants in the study by Eichenberg et al [44] were asked to indicate the perceived impact of health information sources on their health behavior and intentions to use four mental health services. Musiat et al [6] asked their respondents to indicate their views about four mental health services on 12 domains identified through a focus group as important for mental health services (eg, helpfulness).

#### Study Designs

All studies were cross-sectional studies collecting data through self-report measures. One study was an experimental RCT [43], whereas the other three were quasi-experimental surveys [6,42,44].

### Preferred Sources and Use of Mental Health Services

Study findings on preferred mental health services investigated in two of four studies revealed that most participants reported having most often accessed traditional mental health services (eg, GPs, psychologists) and health information websites [42,44]. Individual differences in service usage were investigated in three studies [6,42,44]. In the survey by Klein and Cook [42], the smaller subgroup of “e-preferers” (50/218) has significantly more often indicated to have accessed online counseling (15%) than the larger subgroup of “non e-preferers” (168/218; experience with online counseling: 3.6%). In contrast, the German study revealed [44] that only 14 of 2411 participants (0.6%) reported experience with online counseling; the awareness of online counseling was highest in Web-based health information users, but overall low (11% of n=2411). In the study by Musiat et al [6] 49% of the sample (240/490) reported previous mental health problems and 22% (107/490) indicated current issues; of these persons with prior or current mental health problems, most received a formal diagnosis (60%) and reported experience with help-seeking (85%). Of the 12 domains provided, “helpfulness” was rated as most important for the decision to engage with mental health services. In line with other included Web-based surveys [42,43], this sample consisted mainly of young, well-educated persons with good computer literacy [6].

### Assessment of Study Quality on the Review Level

As shown in Table 2, we evaluated the quality of the studies included in this review as fair, scoring 5 of 10 stars on average. There were issues for the domain “comparability” as the NOS is not designed for scoping reviews. Each study received at least two stars for selection and outcome, but only the RCT [43] received a star for comparability. In addition to limitations, the studies also had several strengths. Musiat et al [6] involved the best described evidence-based measure. However, this could be not emphasized through higher NOS scores. However, the theoretical framework for the assessment of core constructs (attitudes, preference, and technology acceptance) remained largely unclear across included studies.

**Table 2:** The Newcastle-Ottawa scale (NOS) for cross-sectional studies for the assessment of quality of surveys included in this scoping review. This summary does not include single results for subsections.

Study	Selection^a^ (maximum 5 stars)	Comparability (maximum 2 stars)	Outcome (maximum 3 stars)	Total score (maximum 10 stars)
Klein and Cook [42]	***	-	**	*****
Casey et al [43]	****	*	**	*******
Eichenberg et al [44]	***	-	**	*****
Musiat et al [6]	***	-	**	*****

^a^One star for sample size “justified and satisfactory” was only given when the sample was representative or when both a justification for the sample size (eg, power analyses) and a satisfactory sample size were reported.

#### Selection

All studies had justified, sufficient sample sizes. Eichenberg et al [44] had the largest and the only representative sample in this review. Regarding the feasibility of self-developed surveys on indicators of public acceptability, all included studies provided satisfactory information. One of four studies [44] reported pretests. Musiat et al [6] involved a qualitative participatory approach for the measurement development (focus group interviews). Casey et al [43] provided data on internal consistencies of their modified measure. Three studies described response rates and data exclusions [6,42,43]. Klein and Cook [42] combined a self-developed survey and validated measures. However, the self-developed measure concerned main outcomes and thus one star was assigned. Alpha reliabilities were reported for validated measures from original papers [42].

#### Comparability

Only the study by Casey et al [43] was conducted as RCT (one star for control of most important factors). However, as an open access Web-based survey the control of additional factors was questionable. The other studies were noncontrolled observational studies; none of them received a star in this section.

#### Outcome

Each study used self-report measures (assessment of outcome, with one star) and clearly described the used statistical test (one star). In view of the early stage of measurement development for public acceptability assessment, two stars were assigned to all included (pilot) studies.

### Synthesis of Results

We mapped the research findings into two main categories. To answer the research questions, we summarized the study findings on main outcomes for the constructs: (1) perceived helpfulness and (2) intentions to use either e-mental health or traditional mental health services as indicators of public acceptability.

#### Main Findings on Perceived Helpfulness of E-Mental Health Treatment Services

Three of the four studies [6,42,44] compared individual expectations and perceptions about the helpfulness between traditional and e-mental health treatment services. Survey findings indicated that respondents perceived traditional therapy services significantly more helpful for mental health problems than e-mental health treatment services [6,42,44]. Klein and Cook [42] confirmed that “e-preferers” (one-third of the sample) endorsed information websites and Web-based programs without therapist assistance as significantly more helpful than “non e-preferers.” “Non e-preferers” indicated to perceive GPs, psychologists, counselors, telephone counseling services, and prescribed medication as significantly more helpful than “e-preferers.” There was no significant difference in perceived helpfulness of Web-based programs with therapist assistance between e-preference groups, though “non e-preferers” expressed more concerns about Web-based treatments, such as confidentiality issues [42]. Musiat et al [6] showed that e-mental health and mHealth treatment services scored highest on domains such as convenience of access, anonymity, and being free of charge than traditional services. Face-to-face therapy was considered as most acceptable in terms of meeting respondents’ expectations about mental health services in most domains, such as helpfulness, credibility, provision of support, or being appealing. mHealth treatment apps were associated with the lowest acceptability compared with e-mental health programs, face-to-face therapy, and self-help books [6]. Furthermore, both Australian studies revealed that therapist-assisted Web-based programs were perceived as significantly more helpful than Web-based treatments without therapeutic guidance [42,43]. Educational interventions used in the RCT by Casey et al [43] had no significant impact on perceived helpfulness of e-mental health services.

#### Main Findings on Intentions to Use E-Mental Health Treatment Services

Three of the four included studies [6,42,44] compared intentions to use traditional and Web-based treatment services. Results showed that most respondents indicated being more likely to use face-to-face psychotherapy than media-assisted treatments for mental health problems. Guided e-mental health programs were associated with improved intentions to use across studies. For instance, Casey et al [43] revealed significantly lower intentions to use Web-based programs without therapist assistance in comparison to therapist-assisted Web-based programs, information websites, and online counseling. Furthermore, participants who received text-based educational information reported a significant higher likelihood of future use of different e-mental health services (ie, health information websites, online counseling, and programs with or without therapist assistance) in comparison to the control condition. In contrast, no significant effect of film-based educational information on intentions to use e-mental health services was identified [43].

Considering individual differences, Klein and Cook [42] confirmed that “non e-preferers” indicated being more likely to access traditional services provided by psychologists, whereas the far smaller amount of “e-preferers” (one-third of the sample) was more willing to access e-mental health services (ie, online counseling, information websites, and Web-based programs with or without therapist assistance). Conversely, “non e-preferers” reported being likely to use the Internet to seek for health information, but not for treatment. In the German study [44], perceived impact of health information sources corresponded with intentions to use mental health services. Frequent Internet use was associated with intentions to seek mental health advice online. Additional analyses revealed that younger age, being single, higher education level, and household income correlated with intentions to use e-mental health services for psychological distress [44]. Musiat et al [6] showed that respondents who had sought help for mental health problems reported significantly lower intentions to use mHealth treatment apps.

## Discussion

The purpose of this scoping review was to identify the status quo of public preferences, attitudes, and acceptability of e-mental health treatments across different regional contexts. As indicators for the large-scale acceptability, we defined both (1) perceived helpfulness and (2) intentions to use e-mental health treatment services. In the literature, we identified and reviewed four eligible surveys published between 2010 and 2015. The main findings and implications are discussed.

### Summary of Evidence

We identified the following aspects as main findings of included surveys:

Health information websites are widely used and accepted as easy accessible information sources for mental health purposes in everyday life [6,42-44].

Compared with face-to-face treatments, the acceptability of e-mental health treatments services was lower in terms of both the indicators, that is, perceived helpfulness and intentions to use [6,42,44], with the exception of “e-preferers” [42].

Professional support seems to be important for decision making in the context of impending help-seeking intentions. In case of emotional distress, both face-to-face treatments services [6,42,44] and therapist-assisted e-mental health interventions were preferred over unguided Web-based programs [42,43] and self-help books [6]. Perceived helpfulness and intentions to use e-mental health treatments varied across service types provided in the reviewed surveys.

In the RCT [43], neither film- nor text-based educational material has affected perceived helpfulness. Only the text-based material yielded to improved intentions to use Web-based programs, whereas the film intervention was ineffective.

#### Perceived Helpfulness as Indicator for Public Acceptability of E-Mental Health

Most participants surveyed in three of the four studies [6,42,44] reported perceiving traditional interventions as more helpful than e-mental health treatment services. However, Musiat et al [6] argued that low “e-awareness” together with the questionnaire design (comparisons between established and novel approaches) might have led participants to believe that face-to-face psychotherapy was a “benchmark.” Thus, perceptions of helpfulness of e-mental health treatments may have been biased toward negative assessments [6]. Nonetheless, it could be also argued that not the delivery mode (Web-based or face-to-face) is essential, but therapeutic assistance is the key for large-scale acceptability. This argument is supported by the observation that assessments of perceived helpfulness were generally informed for therapist-assisted interventions across included studies (both guided e-mental health and face-to-face treatment services) in comparison to unguided services [6,42-44], including conventional self-help books [6]. The role of e-mental health literacy in attitudes remains unclear. The provision of educational information about e-mental health [43] was ineffective in influencing perceptions of helpfulness across four e-mental health types (ie, guided and unguided Web-based programs, online counseling, and information websites).

#### Intentions to Use as Indicator for Public Acceptability of E-Mental Health

Consistent with the findings for the acceptability indicator “perceived helpfulness,” three of the four included studies (comparing Web-based and traditional services) suggested an overall higher likelihood to use face-to-face therapy than e-mental health treatments in case of emotional distress [6,42,44]. In addition, the RCT by Casey et al [43], which compared e-mental health services, revealed the highest likelihood of future for therapist-assisted Web-based programs. This conclusion is also in line with the results for perceived helpfulness. Interestingly, only the text-based material was associated with improved assessments regarding intentions to future use e-mental health, since the (identical) film-based intervention was shown being ineffective [43].

Moreover, sociodemographic group differences in intentions to use e-mental health treatments were only found in the German survey using a representative sample [44]. Internet users appeared generally more open to use e-mental health services such as information websites and online counseling in case of emotional distress [42,44]. This indicated the relevance of familiarity with new media and e-awareness for acceptability [6]. However, the evidence base for these correlative associations (as identified in this scoping review) is too small to derive definitive conclusions.

Another point worthy of note is the coherence of lower public acceptability of e-mental health in comparison to face-to-face services identified across included studies, although data were collected in different countries varying basically in their stage of implementation of e-mental health into routine care.

### Comparisons With Previous Work

Several findings identified in this scoping review are consistent with previous research. For instance, there was coherence between assessments of perceived helpfulness and intentions to use across all studies included in this review. Oh et al [27] have also demonstrated that perceived helpfulness of e-mental health services was associated with improved acceptability and intention to use Web-based self-help websites in young Australians. Most studies targeting attitudes or indicators of acceptability of e-mental health treatments surveyed not the samples of the general population, but specific populations such as adolescents [27-30] or patients [31,32]. This limitation is important because these studies are not directly applicable to public acceptability research. Selective samples are an issue for the generalizability of study findings in e-mental health research [20]. In contrast to the three Web-based surveys [6,42,43] included in this review, the representative sample from Germany [44] revealed sociodemographic differences in user experience, preferred services, awareness, and intentions to use e-mental health. The study by Eichenberg et al [44] has shown that experience with e-mental health was associated with improved intentions to use such services [44]. This result is in line with another Australian study by Gun et al [39] that has also confirmed that both professionals and laypersons who have previously used e-mental health services were more likely to evaluate Web-based treatments as acceptable. However, it should also be noted that the Australian survey by Klein and Cook [42] indicated lower acceptability of e-mental health compared with traditional mental health treatments despite the familiarity of participants with e-mental health services, such as information websites and Internet-delivered interventions. Framework for technology adoption also indicated that experience or familiarity with innovative technology can inform their acceptance [20,22-24]. The UTAUT [22,23] suggested habits or experience as important moderator of intentions to use a technology. Thus, the regional context of included studies and stage of implementation of e-mental health service into health care should be considered for the interpretation of results. In contrast to the English [6] and the two Australian Web-based surveys [42,43] included in this scoping review, the German survey [44] was conducted in the context of a health care system being at an early stage of e-mental dissemination in public mental health care [1,17]. The representative German sample consisted of a relatively high number of respondents who indicated being nonusers of the Internet or infrequent users of new media and computer technologies; furthermore, Eichenberg et al [44] identified both low usage and awareness of online counseling in the German general population in 2010 (year of data collection). These contextual factors can be interpreted according to the diffusion of innovation theory [22], in which familiarity with the innovation and time for adoption play crucial roles for dissemination. Thus, the rather low public acceptability observed internationally raises doubt if the mere exposure with e-mental health services in primary care is the “condition sine qua non” for improved public acceptability.

Sociodemographic differences in help-seeking intentions were identified by Crisp and Griffiths [40]. The authors have demonstrated that Australians who were interested in participating in e-mental health programs were more likely female, “older” in average than most respondents, higher educated, divorced, and reported a history of mental health problems (depressive symptoms) as well as lower self-stigma in comparison to individuals who denied attending an e-mental health intervention. These findings are partly in line with sociodemographic differences found in the representative sample, such as education level [44]. However, two Web-based studies included in this scoping review showed conflicting results. For instance, Klein and Cook [42] showed that “e-preferers” were more likely to express self-stigma than “non e-preferers.” Furthermore, Musiat et al [6] revealed that persons with a history of mental health problems expressed the lowest acceptability for mHealth treatment services. This scoping review was also not designed to clarify in which cases experience with traditional mental health care (and history of mental disorders) is positive or negative for the individual acceptability of e-mental health treatments. In the two included studies [6,42], relatively high numbers of participants reported experience with mental health care services and seeking help. It thus appears that sample characteristics and recruitment contexts are crucial to explain such inconsistencies. Nevertheless, due to methodological heterogeneity the results of these studies [6,40,42] should be compared cautiously.

Unfortunately, Casey et al [43] have not measured the mental health status or previous service experience with mental health care among respondents as potential factors for mostly nonsignificant findings of their RCT (especially in terms of the ineffective film-based material). In contrast to this finding, another RCT from Germany conducted by Ebert et al [45] with depressive primary care patients showed that a film intervention has yielded to significantly improved acceptability of an e-mental health treatment in comparison to the control condition. However, the film in the clinical trial [45] was with a duration of 7 minutes longer than the film used in the RCT by Casey et al [43]. In addition, the language, target group, setting, and content of the educational intervention differed across both RCTs. Therefore, it is recommended that future research should systematically vary provision modes or information material to identify most effective features and address both facilitators and barriers in terms of technology use. In accordance with a review by Lal and Adair [3], concerns reported in included studies [6,42,43] were mostly related to data security issues. These concerns could function as barriers to use e-mental health services and their impact thus needs further clarification.

Taken together, the (not directly targeted) question whether e-awareness and e-mental health literacy are key facilitators for the acceptability of e-mental health and mHealth treatment services [6] could not be answered in this scoping review (not our focus) and should be hence addressed in upcoming reviews.

### Limitations

This scoping review has several limitations. On the study level, the external validity of three of four studies [6,42,43] using self-report measures in Internet-based open access surveys is questionable. Web-based data collection modes resulted in selective samples, which mainly comprised young, well-educated, and female respondents. Conversely, it can be argued that these sample characteristics are common features of e-mental health service users and that selection bias is a general problem in e-mental health research [17,20]. Furthermore, we have also identified sources of heterogeneity in terms of varying ways of operationalization of psychological constructs such as attitudes. Moreover, exploratory statistical analyses undertaken in the three included quasi-experimental studies involved subsample comparisons (sociodemographic differences in intentions to future use e-mental health) between unequally sized groups [6,42,44]. In addition, all included studies operationalized intentions to use mental health services in the (assumed) absence of mental health problems. However, Musiat et al [6] revealed that nearly one-fourth of their sample indicated current mental health problems. On the review level, the search strategy we used may have led to incomplete retrieval of records, which is an important point to consider for systematic reviews. For instance, we searched databases using “e-mental health” as a non-MeSH term. In addition, despite the existence of checklists for reporting results of e-health studies [46], terminology is inconsistent in the e-health research literature [47]. In this sense, various keywords for similar service types are an obstacle for reviews. Furthermore, we used the NOS for the quality assessment despite potential reliability issues [48]. A final point to consider is that the clear majority of eHealth publications reports positive conclusions [49]. This indicates a risk for publication bias, which we have not examined in this review. Concerning both methodological issues of included studies and poor evidence base for public acceptability, findings presented in this review should be interpreted cautiously. Nonetheless, to our knowledge, this is the first review targeting indicators of public acceptability of e-mental health treatments.

### Implications for Practice and Research

Considering the popularity of health information websites [42,44] on the one hand and slow implementation of e-mental health into health care systems on the other, it appears surprising that both potential facilitators and barriers of public acceptability of these innovative treatment services are still understudied [6]. All survey included in this scoping review were pilot studies with diverse strengths and limitations. This scoping review derived several implications. Considering neutral or even negative attitudes toward e-mental health treatment services identified across studies, promoting “e-awareness” among citizens and professionals has been suggested as a promising strategy for strengthening their public acceptability in the long run [6,43]. Promoting e-health literacy is essential since lacking computer or new media competencies hinder service users from effectively searching and using health information on the Internet [50]. Considering the impact of e-mental health literacy on help-seeking behavior in general, it should be considered that the evidence base is too small to derive more precise recommendations [50,51]. In addition, it remains unclear how information material should be designed to improve attitudes toward e-mental health treatments [43]. On the basis of the heterogeneity of study findings on public acceptability, it can be concluded that targeting the general population and using questionnaires (risk of biased assessments based on vague definitions of laypersons as respondents) involves many uncertainties. Hence, studies using qualitative methods to survey preferences and attitudes toward e-mental health in specific target groups can complement quantitative research. For instance, studies in e-mental health research have used focus group interviews [6,28,29]. Such qualitative studies in university settings have shown concerns about data security and confidentiality that can turn out as key obstacle to use e-mental health services [52,53].

Furthermore, this scoping review has identified opportunities to improve the validity of self-developed measures. All studies included in this scoping review appeared to have grounded their self-developed measures mainly on research evidence, without referring to applicable theoretical frameworks, such as the diffusion of innovation theory [22] and the UTAUT [23,24]. Nonetheless, it is apparent that such frameworks need to be adapted for e-mental health research. Recent studies [54-56] have already shown opportunities for transferring the UTAUT to eHealth uptake research. These adaptions can help determine key factors for public acceptability of e-mental health. However, it should also be considered that the complexity of health care is associated with limitations for using the UTAUT for this purpose [26]. As complement research strategy, theory-led, participatory frameworks such as the person-based approach [21] can add further value toward a deeper understanding of individual needs in the context of digital health interventions through using qualitative or mixed methods. Overall, having a toolkit of such evidence-based approaches provides further flexibility in the emerging field of public acceptability of e-mental health treatments. However, collaborative interdisciplinary approaches require more time and efforts than quantitative surveys, and thus mixed methods are promising strategies.

Given the significance of innovative treatment strategies for public health, it can be anticipated that the evidence base for public acceptability and attitudes toward e-mental health services will grow in the next years. Future reviews could thus address both questions left open in this scoping review and compare study findings on acceptability of e-mental health between different groups of adopters, such as patients and clinicians [57] or laypersons and health professionals. For instance, Carper et al [33] showed rather negative attitudes toward e-mental health treatments among help-seeking persons, whereas surveyed health professionals tended to report neutral views. Such discrepancies appear interesting for further investigations. Furthermore, individual differences between different groups of adopters such as “e-preferers” [6] should be also further clarified. In essence, an understanding of facilitators and barriers of public acceptability of e-mental health treatment provides orientation to not get lost in e-mental health implementation.

### Conclusions

This scoping review has explored both perceived helpfulness and intentions to use as indicators of public acceptability of e-mental health treatment services. Findings of four included pilot studies have indicated professional support as key facilitator of public acceptability of traditional and e-mental health services. While e-awareness was also suggested as a factor improving the uptake of e-mental health, associations with intentions to use digital interventions were rather anecdotal. Thus, the impact of e-mental health literacy and informed decision-making on e-mental health uptake should be further explored. However, the small evidence base and methodological issues of included surveys such as Web-based data collection or unclear theoretical framework underpinning self-developed measures left several questions open. To assess and understand the complex field of public acceptability of e-mental health treatments, consistent operationalization of constructs in future studies is required.

## Appendix

Multimedia Appendix 1 Poster presentation of the previous review at the 4th ESRII conference.

Multimedia Appendix 2 Poster presentation of the scoping review at the 30th EHPS conference.

## Abbreviations

CBT: cognitive behavior therapy
e-mental health: electronic mental health
GP: general practitioner
iCBT: Internet-based cognitive behavior therapy
IT: information technology
MeSH: medical subject headings
m-mental health: mobile mental health
NOS: Newcastle-Ottawa scale
PRISMA: preferred reporting items for systematic reviews and meta-analyses
RCT: randomized controlled trial
TAM: technology acceptance model
UTAUT: unified theory of acceptance and use of technology
