# Supplementary material for: Public Acceptability of E-Mental Health Treatment Services for Psychological Problems: A Scoping Review
Source: JMIR Ment Health. 2017 Apr 3;4(2):e10. doi: 10.2196/mental.6186 (PMC5394261; doi:10.2196/mental.6186)
Supplement: Multimedia Appendix 1 [file mental_v4i2e10_app1.pdf]

4th Conference of the European Society for Research on Internet Interventions (ESRII) in Bergen, Norway| Poster Presentation | 23th September 2016

Attitudes toward e-mental health services in the general population: a systematic review

Jennifer Apolinário-Hagen, Jessica Kemper & Carolina Stürmer
FernUniversität in Hagen

Contact: Dr. Jennifer Apolinário-Hagen, FernUniversität in Hagen / University of Hagen, Dep. of Health Psychology, 58084 Hagen, Germany | **E-Mail**: jennifer.apolinario-hagen@fernuni-hagen.de

BACKGROUND

– Regarding potential **(psychological) barriers** to dissemination and **implementation** of **e-mental-health** services into healthcare, **attitudes and preferences** of help-seeking individuals are considered having predictive value for the large-scale acceptability [1,2].

– However, **research on public acceptability of e-mental health** is a relatively novel field and the evidence base from surveys appears fragmentary.

Objective

Thus, this review aims at identifying public views on online self-help services.

METHODS

- **Systematic search of the literature** through electronic databases (e.g. Medline/Pubmed; PsycINFO, PsychArticels)
- **Inclusion criteria:** non-clinical surveys on public views of e-mental health (all age groups were included), published between 01/2005 and 12/2015 in peer-reviewed journal in German or English language
- **Exclusion oriteria:** e.g. clinical trials, surveys targeting patients´ or professionals´ views or narrowed/specific types of e-health services
- **Keywords**: incl. „e-mental health" and „online self-help" + „acceptability"; „attitudes"; „preference"; „perceptions"
- **Indicators of acceptability**: „perceived helpfulness"; „likelihood of future use" (UTAUT framework [3])

RESULTS. Summary of Key Findings (n = 24 publications *)

Study Characteristics

– In total, we identified **24 eligible studies** with sample sizes ranging from 23 to 4.758.

– Participants were between eight and 95 years of age. Data was collected in North America, Australia and Europe (England, Ireland, Germany)

– Most studies applied self-report measures (n=19 studies), some qualitative methods (interviews, n = 4, focus groups and interviews, n = 1)

Key Results

– Overall, results indicated **preferences to seek professional help provided face-to-face over e-mental health services.**

– With respect to e-mental health services, **professional support** and **immediate feedback** were preferred features.

– **Motives for usage** of e-mental health included **anonymity and flexibility** in terms of time and location.

– **Likelihood of future** using digital services was found being associated with **e-awareness** and in some samples with **sociodemographic factors** (albeit with mixed findings). Nonetheless, the role of individual background on e-attitudes, respectively e-preference remains still largely unclear.

CONCLUSIONS

Public views on eMental Health Treatments – „nice to have" or „must have"?

– Currently, the evidence base on attitudes towards e-mental health services is limited (black box).

– Future studies should address this gap, e.g. by using mixed methods for measure development.

Limitations

– **Study level:** Lack óf both validated measures and theory-lead rationales (e.g. definition of attitudes); indications for low e-awareness and eMental health literacy in some countries (e.g. Germany)

– **Review level**: e.g. keywords did not include „mHealth" (use of „eMental Health" as umbrella term)

Implications: Psychoeducational information might have an positive impact on attitudes towards e-mental health services, in terms of the likelihood of future use in case of psychological problems [4]

Abb. 1. *Basic Technology Acceptance Models* [3]

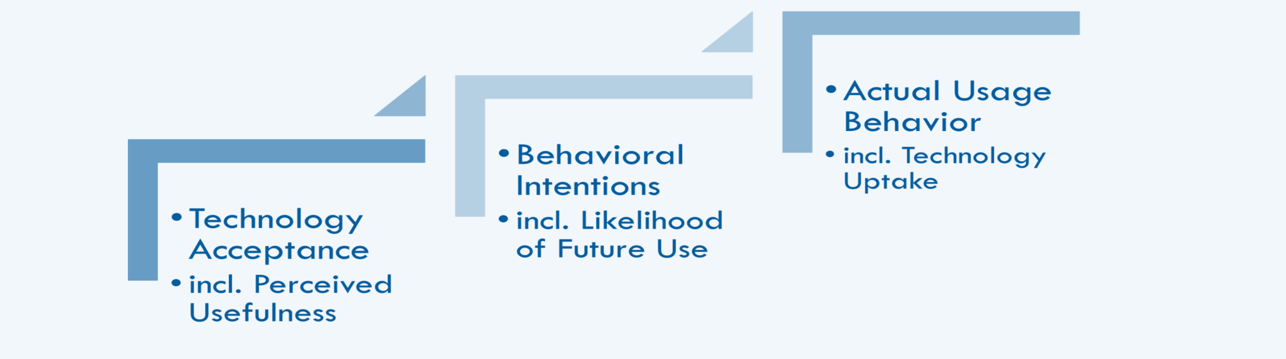

References. (* articles included in this review)

[1] * Musiat, P., Goldstone, P., & Tarrier, N. (2014). Understanding the acceptability of e-mental health - attitudes and expectations towards computerised self-help treatments for mental health problems. BMC psychiatry, 14(1). doi: 10.1186/1471-244X-14-109 [2] * Klein, B., & Cook, S. (2010). Preferences for e-mental health services amongst an online Australian sample. Electronic Journal of Applied Psychology, 6(1), 27-38. doi: 10.7790/ejap.v6i1.184
[3] Venkatesh,V., Morris, M.G., Davis, G.B., & Davis, F.D. (2003). User acceptance of information technology: toward a unified view. MIS Quarterly, 27(3), 425-478.
[4] * Casey, L.M., Joy, A., & Clough, B.A. (2013). The Impact of Information on Attitudes Toward E-Mental Health Services. Cyberpsychology, Behavior, and Social Networking, 16(8), 593-598. doi: 10.1089/cyber.2012.0515.
*Bradford, S., & Rickwood, D. (2014). Adolescent's preferred modes of delivery for mental health services. Child and Adolescent Mental Health, 19(1), 39-45. doi: 10.1111/camh.12002
*Casey, L.M., Joy, A., & Clough, B.A. (2013). The Impact of Information on Attitudes Toward E-Mental Health Services. Cyberpsychology, Behavior, and Social Networking, 16(8), 593-598. doi: 10.1089/cyber.2012.0515
*Choi, I., Sharpe, L., Li, S., & Hunt, C. (2015). Acceptability of psychological treatment to Chinese- and Caucasian-Australians: Internet treatment reduces barriers but face-to-face care is preferred. Social psychiatry and psychiatric epidemiology, 50(1), 77-87. doi: 10.1007/s00127-014-0921-1
*Crabb, R.M, Rafie, S., & Weingardt, K.R. (2012). Health-related internet use in older primary care patients. Gerontology, 58(2), 164-170. doi: 10.1159/000329340
*Crisp, D.A., & Griffiths, K.M. (2014). Participating in online mental health interventions: Who is most likely to sign up and why? Depression Research and Treatment, 1-11. doi: 10.1155/2014/790457
*Eichenberg, C., & Aden, J. (2015). Onlineberatung bei Partnerschaftskonflikten und psychosozialen Krisen. Multimodale Evaluation eines E-Mail-Beratungsangebots. Psychotherapeut, 60(1), 53-63. doi: 10.1007/s00278-014-1092-y
*Eichenberg, C., & Brähler, E. (2013). Internet als Ratgeber bei psychischen Problemen. Bevölkerungsrepräsentative Befragung in Deutschland. Psychotherapeut, 58(1), 63-72. doi: 10.1007/s00278-012-0893-0
*Ellis, L.A., Collin, P., Hurley, P.J, Davenport, T.A., Burns, J.M, & Hickie, I.B. (2013). Young men's attitudes and behaviour in relation to mental health and technology: implications for the development of online mental health services. BMC psychiatry, 13:119. doi: 10.1186/1471-244X-13-119
*Elsegood, K., & Powell, D. (2008). Computerised cognitive-behaviour therapy (cCBT) and older people: A pilot study to determine factors that influence willingness to engage with cCBT. Counselling and Psychotherapy Research, 8(3), 189-192. doi: 10.1080/14733140802163914
*Gun, S.Y., Titov, N., & Andrews, G. (2011). Acceptability of Internet treatment of anxiety and depression. The Royal Australian and New Zealand College of Psychiatrists, 19(3), 259-264. doi: 10.3109/10398562.2011.562295
*Horgan, A., & Sweeney, J. (2010). Young students' use of the Internet for mental health information and support. Journal of Psychiatric and Mental Health Nursing, 17, 117–123. doi: 10.1111/j.1365-2850.2009.01497.x
*King, R., Bambling, M., Lloyd, C., Gomurra, R., Smith, S., Reid, W., & Wegner, K. (2006). Online counselling: The motives and experiences of young people who choose the Internet instead of face to face or telephone counselling. Counselling and Psychotherapy Research, 6(3), 169-174. doi: 10.1080/14733140600848179
*Klein, B., & Cook, S. (2010). Preferences for e-mental health services amongst an online Australian sample. Electronic Journal of Applied Psychology, 6(1), 27-38. doi: 10.7790/ejap.v6i1.184
*Leach, L.S., Christensen, H., Griffiths, K.M, Jorm, A.F., & Mackinnon, A.J. (2007). Websites as a mode of delivering mental health information: perceptions from the Australian public. Social psychiatry and psychiatric epidemiology, 42(2), 167-172. doi: 10.1007/s00127-006-0138-z
*Mar, M.Y., Neilson, E.K., Torchalla, I., Werker, G.R., Laing, A., & Krausz, M. (2014). Exploring e-Mental Health Preferences of Generation Y. Journal of Technology in Human Services, 32(4), 312-327. doi: 10.1080/15228835.2014.943457
*McClay, C., Waters, L., Schmidt, U., & Williams, C. (2014). A Survey of Attitudes towards Computerized Self-Help for Eating Disorders within a Community-Based Sample. Behavioural and Cognitive Psychotherapy, 44(1), 65-78. doi: 10.1017/S1352465814000484
*Mitchell, N., & Gordon, P.K. (2007). Attitudes Towards Computerized CBT for Depression Amongst a Student Population. Behavioural and Cognitive Psychotherapy, 35(4), 421-430. doi:10.1017/S1352465807003700
*Moessner, M., & Bauer, S. (2012). Online counselling for eating disorders : Reaching an underserved population? Journal of Mental Health, 21(4), 336-345. doi: 10.3109/09638237.2011.643512
*Mohr, D.C., Siddique, J., Ho, J., Duffecy, J., Jin, L., & Fokuo, J.K. (2010). Interest in behavioral and psychological treatments delivered face-to-face, by telephone, and by internet. Annals of behavioral medicine, 40(1), 89-98. doi: 10.1007/s12160-010-9203-7
*Musiat, P., Goldstone, P., & Tarrier, N. (2014). Understanding the acceptability of e-mental health - attitudes and expectations towards computerised self-help treatments for mental health problems. BMC psychiatry, 14(1). doi: 10.1186/1471-244X-14-109
*Oh, E., Jorm, A.F., & Wright, A. (2009). Perceived helpfulness of websites for mental health information: a national survey of young Australians. Social Psychiatry and Psychiatric Epidemiology, 44, 293–299. doi: 10.1007/s00127-008-0443
*Stallard, P., Velleman, S., & Richardson, T. (2010). Computer Use and Attitudes Towards Computerised Therapy Amongst Young People and Parents Attending Child and Adolescent Mental Health Services. Child and Adolescent Mental Health, 15(2), 80-84. doi: 10.1111/j.1475-3588.2009.00540.x
*Tsan, J.Y., & Day, S.X. (2007). Personality and Gender as Predictors of Online Counseling Use. Journal of Technology in Human Services, 25(3), 39-55. doi: 10.1300/J017v25n03_03
*Young, K.S. (2005). An Empirical Examination of Client Attitudes Towards Online Counseling. CyberPsychology & Behavior, 8(2), 172-177. doi: 10.1089/cpb.2005.8.172
