# Supplementary material for: Public Acceptability of E-Mental Health Treatment Services for Psychological Problems: A Scoping Review
Source: JMIR Ment Health. 2017 Apr 3;4(2):e10. doi: 10.2196/mental.6186 (PMC5394261; doi:10.2196/mental.6186)
Supplement: Multimedia Appendix 2 [file mental_v4i2e10_app2.pdf]

30th Conference of the EHPS/DHP "Behaviour Change: Making an impact on health and health services" l 23rd - 27th of August, 2016
Aberdeen, Scotland l Interactive Posters (T1) l Friday, 26th of August, 2016 l „eHealth and mHealth 3“ l Chair: Robert West

Public Views on E-Mental Health Services: A systematic Review of the Current Evidence.

Jennifer Apolinário-Hagen

FernUniversität in Hagen, Germany ¥

¥ FernUniversität in Hagen, Institute for Psychology, Department of Health Psychology, 58084 Hagen, Germany | eMail: jennifer.apolinario-hagen@fernuni-hagen.de

BACKGROUND

- **Common mental health problems** are a burden for European healthcare systems.
- However, **individuals with mental health problems face different barriers to access mental healthcare**, such as waiting time, lacking health literacy or stigmatised beliefs.
- Given both **"Dr. Google" as common informal health advisor** and limited capacities of (low-treshold) traditional face-to-face services in healthcare, **e-mental health services** are suggested as viable option to **inform the access to professional help**.
- To overcome barriers to care on a large-scale via innovative technologies, though, **knowledge about the public acceptability of e-mental health** is required.

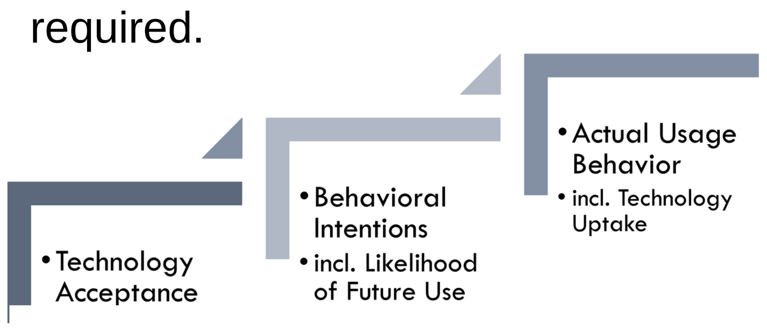

Technology Acceptance Models (e.g. UTAUT [1])

OBJECTIVE:

- To explore the current evidence base on both public views and attitudes toward e-mental-health

RESULTS

- Of 63 screened abstracts, n = 4 papers were included in this review.
- **Sample sizes** ranged from n = 217 to 2.411 persons, aged between 14 and 95 years. Data stem from England [5], Australia [2,3] and Germany [4] .
- **Methodology** varied across studies; all used self-developed surveys (n = 3 online surveys [2,3,5]; n =1 CAPI panel [4]) One study applied mixed methods to measure development [5]

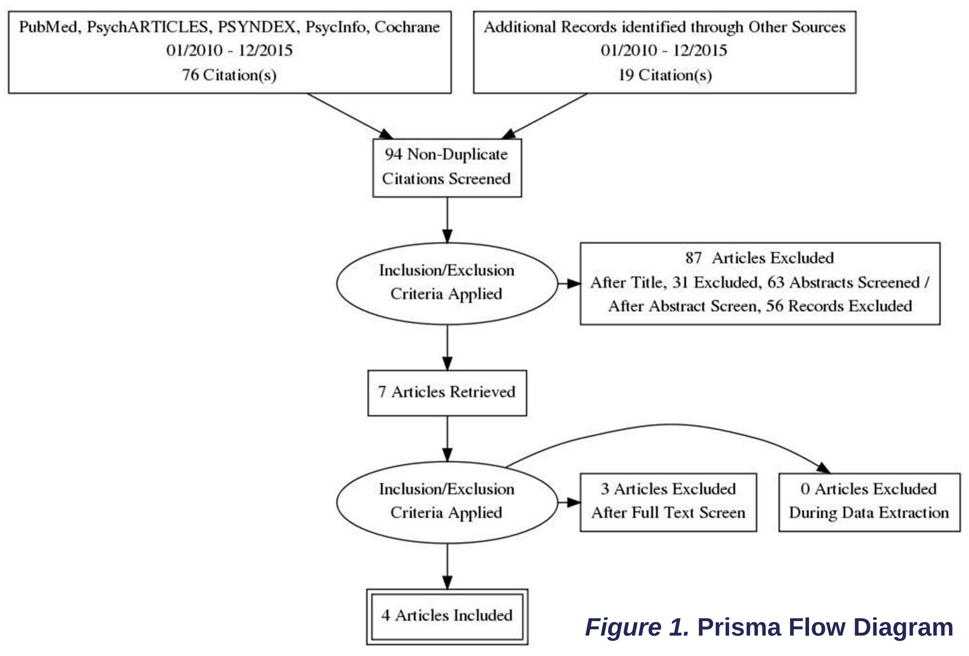

Figure 1. **Prisma Flow Diagram**

KEY FINDINGS:

- Results indicated **type-specific differences in preferences** to mental health services: **Preference to seek help traditional face-to-face services** over eHealth and mHealth services in case of emotional distress was shown.
- **Lowest acceptability** was identified for **mHealth** and **unguided online therapy**
- Despite neutral to negative views on (unguided) e-mental health services reported across studies, **e-health literacy and e-awareness** tended to be associated with improved acceptability in terms of **willingness to future use online self-help**.

Table 1.

Literature Review on Public Views on E-Mental Health: Summary of Study Characteristics, Outcomes, and Main Findings.

Study	Design	Aim/s	Sample	Method and Measures	Main Findings
Klein & Cook (2010). [2]	Cross-sectional online survey	To identify differences between "e-preferers" and "non e-preferers" regarding the perceived helpfulness and likelihood of future using mental health services	Online sample (N = 218) of the Australian general population. ♀ 75.7 % Age range = 18 - 80 years; *M* = 36.6 (*SD* = 14.5) * "e-prefers" (n = 50); "non e-prefers" (n = 168) * 63.9 % with mental service experience	Self-developed online survey and validated personality measures - "(non) e-preference" (grouping condition) - Perceived helpfulness of 11 mental health services - Likelihood of future using mental health services	- Preference to traditional over e-mental health services (77.1 %). - Higher willingness of "e-preferers" to use and assess e-mental health as helpful - "non e-preferers" were more concerned about confidentiality issues - "e-preferers" scored higher on self-stigma than "non e-preferers"
Casey, Joy & Clough (2013). [3]	Cross-sectional online RCT	To determine the impact of information on attitudes toward different e-mental health services	Online sample (N = 217) of the Australian general population. ♀ 78 % Age range = 17 - 60 years;, *M* = 29.7 (*SD* = 11.9) * educational information groups: text (n = 66), film (n = 72), control (n = 70)	Self-developed online survey (modified version of [2]). - Perceived helpfulness of four e-mental health services - Likelihood of future using e-mental health services Random assignment of respondents to one of three conditions	- Preference toward using e-mental health services with therapist assistance - The likelihood of using e-mental health services was improved in the text condition group, but not in the film condition group - Neither the text- nor video-based information affected the perceived helpfulness of e-mental health in comparison to the control condition
Eichenberg, Wolters & Brähler (2013). [4]	Cross-sectional panel survey (CAPI)	To explore public media use, perceived impact of health information sources, and willingness of future using e-mental health	Representative sample (N = 2.411) of the German general population. ♀ 53.2 % Age range = 14 - 90 years;, *M* = 51.0 (*SD* = 18.6) years * 41 % never used computers	Self-developed survey (pre-test with n = 67). - Preferred information sources / their impact on health behaviour - Use of and willingness to use psychological online counselling, and media-assisted in comparison to face-to-face services	- Preference toward using traditional to e-mental health services - Previous use of the internet for health information was associated with a higher willingness to use online counselling - Socio-demographic data (e.g. age, gender, education) and internet usage corresponded with readiness to use e-mental health
Musiat, Goldstone & Tarrier (2014). [5]	Cross-sectional online survey	To explore the acceptability of e- and m-mental health services in comparison to traditional services	Online sample (N = 490) of the English general population. ♀ 78.2 % Age range =18 - 78 years;, *M* = 26.7 (*SD* = 8.9) * 49 % with a history of mental problems	Self-developed survey (grounded on focus group of service users). - Expectations and acceptability: features of mental health services - Perceived benefits, concerns and likelihood of future using e-mental health and m-health in comparison to traditional services	- Preference to traditional over e-mental health and m- health apps - Traditional face-to-face treatments were most likely to meet respondents´ expectations in most important aspects (e.g. helpfulness, credibility) - Lowest acceptability was expressed for m-health apps as provision mode

Note. ♀ = female gender; Abbreviations: CAPI = computer-assisted personal interview; e-mental health = electronic mental health; m-health apps = mobile mental health applications; RCT = randomized controlled trial.

METHODS

- **Systematic review:** Literature search through electronic databases (e.g. Medline)
- **Inclusion criteria:** Surveys targeting acceptability, expectations, preferences and/or attitudes toward e-mental health treatments in the general population, published in peer-reviewed English journals between 01/2010 and 12/2015.
- **Exclusion criteria:** Clinical trials or surveys with narrowed scope (e.g. specific target groups or of e-mental health services)
- **Search terms:** incl. e-mental health; attitude; preference; online self-help; iCBT

CONCLUSIONS

- Currently, the evidence base on public acceptability of e-mental health is very small.
- Perceived helpfulness and likelihood of future use were indicators of IT acceptance.

Limitations:

- **Lack of theory-lead rationales** in self-developed surveys (e.g. defining attitudes)
- **Low e-mental health literacy** and **e-awareness** in surveyed (selective) samples.

Implications:

- Future studies should consider applying the UTAUT [1] framework to inform the comparability of self-report measures on public e-mental health acceptance.

References: [1] Venkatesh,V., Morris, M. G., Davis, G. B. & Davis, F. D. (2003). User acceptance of information technology: toward a unified view. MIS Quarterly, 27(3), 425-478. [2] Klein, B. & Cook, S. (2010). Preferences for e-mental health services amongst an online Australian sample. Electronic Journal of Applied Psychology, 6(1), 27-38. doi: 10.7790/ejap.v6i1.184 [3] Casey, L. M., Joy, A. & Clough, B. A. (2013). The Impact of Information on Attitudes Toward E-Mental Health Services. Cyberpsychology, Behavior, and Social Networking, 16(8), 593-598. doi: 10.1089/cyber.2012.0515. [4] Eichenberg, C., Wolters, C. & Brähler, E. (2013) The Internet as a mental health advisor in Germany: results of a national survey. PLoS ONE 8(11): e79206. doi:10.1371/journal.pone.0079206. [5] Musiat, P., Goldstone, P. & Tarrier, N. (2014). Understanding the acceptability of e-mental health - attitudes and expectations towards computerised self-help treatments for mental health problems. BMC Psychiatry, 14(1). doi: 10.1186/1471-244X-14-109

*** for the poster abstract, please see: Apolinário-Hagen J. Public views on e-mental health services –a systematic review of the current evidence. European Health Psychologist. 2016;18:981.
